# Partitioning the metabolic scope: the importance of anaerobic metabolism and implications for the oxygen- and capacity-limited thermal tolerance (OCLTT) hypothesis

**DOI:** 10.1093/conphys/cow019

**Published:** 2016-06-03

**Authors:** Rasmus Ejbye-Ernst, Thomas Y. Michaelsen, Bjørn Tirsgaard, Jonathan M. Wilson, Lasse F. Jensen, John F. Steffensen, Cino Pertoldi, Kim Aarestrup, Jon C. Svendsen

**Affiliations:** 1 Department of Chemistry and Bioscience, Faculty of Engineering and Sciences, Aalborg University, Aalborg, Denmark; 2 Marine Biological Section, Department of Biology, University of Copenhagen, Helsingør, Denmark; 3 Molecular Eco-physiology, Interdisciplinary Center of Marine and Environmental Research (CIIMAR), University of Porto, Porto, Portugal; 4 Department of Biology, Wilfrid Laurier University, Waterloo, Ontario, Canada; 5 Fisheries and Maritime Museum, Esbjerg, Denmark; 6 Aalborg Zoo, Aalborg, Denmark; 7 National Institute of Aquatic Resources, Technical University of Denmark (DTU), Silkeborg, Denmark; 8 National Institute of Aquatic Resources, Technical University of Denmark (DTU), Charlottenlund, Denmark

**Keywords:** Aerobic metabolic scope, anaerobic metabolism, oxygen- and capacity-limited thermal tolerance (OCLTT), sea bream (*Sparus aurata*), trade-off, Trinidadian guppy (*Poecilia reticulata*)

## Abstract

Ongoing climate change is predicted to affect the distribution and abundance of aquatic ectotherms owing to increasing constraints on organismal physiology, in particular involving the metabolic scope (MS) available for performance and fitness. The oxygen- and capacity-limited thermal tolerance (OCLTT) hypothesis prescribes MS as an overarching benchmark for fitness-related performance and assumes that any anaerobic contribution within the MS is insignificant. The MS is typically derived from respirometry by subtracting standard metabolic rate from the maximal metabolic rate; however, the methodology rarely accounts for anaerobic metabolism within the MS. Using gilthead sea bream (*Sparus aurata*) and Trinidadian guppy (*Poecilia reticulata*), this study tested for trade-offs (i) between aerobic and anaerobic components of locomotor performance; and (ii) between the corresponding components of the MS. Data collection involved measuring oxygen consumption rate at increasing swimming speeds, using the gait transition from steady to unsteady (burst-assisted) swimming to detect the onset of anaerobic metabolism. Results provided evidence of the locomotor performance trade-off, but only in *S. aurata*. In contrast, both species revealed significant negative correlations between aerobic and anaerobic components of the MS, indicating a trade-off where both components of the MS cannot be optimized simultaneously. Importantly, the fraction of the MS influenced by anaerobic metabolism was on average 24.3 and 26.1% in *S. aurata* and *P. reticulata*, respectively. These data highlight the importance of taking anaerobic metabolism into account when assessing effects of environmental variation on the MS, because the fraction where anaerobic metabolism occurs is a poor indicator of sustainable aerobic performance. Our results suggest that without accounting for anaerobic metabolism within the MS, studies involving the OCLTT hypothesis could overestimate the metabolic scope available for sustainable activities and the ability of individuals and species to cope with climate change.

## Abstract (precis)

Climate change is predicted to affect the aerobic capacity available for sustainable performances and fitness in fishes. This study reveals trade-offs between aerobic and anaerobic components of swimming performance and metabolic scope, and highlights the possibility of overestimating sustainable aerobic performances of fishes in relation to climate change.

## Introduction

Effects of climate change (e.g. increased temperature, ocean acidification and hypoxia) are predicted to have profound effects on the physiology of aquatic ectotherms, including fishes (Pörtner and Peck, 2010; IPCC, 2014; Deutsch *et al.*, 2015). This has raised conservation concerns for the persistence of fish populations and increased the interest in developing predictive models for effects of climate change on different species (Jørgensen *et al.*, 2012; Farrell, 2016). The oxygen- and capacity-limited thermal tolerance (OCLTT) hypothesis uses the metabolic scope (MS) to derive a range of tolerable temperatures (thermal window) of organisms with an optimal temperature for oxygen supply to sustain life, including growth, foraging, migration and reproduction (Pörtner and Farrell, 2008; Pörtner and Peck, 2010; Holt and Jørgensen, 2015; Motyka *et al.*, 2016; Verberk *et al.*, 2016). The MS is defined as the difference between the maximal metabolic rate ($\dot{M}$O_2max_) and standard metabolic rate ($\dot{M}$O_2stand_). Importantly, OCLTT is concerned solely with the aerobic component of MS (Pörtner and Farrell, 2008; Eliason *et al.*, 2011; Clark *et al.*, 2013), essentially making the assumption that any anaerobic component is insignificant. Anaerobic metabolism within the MS is important because it depletes substrates (e.g. glycogen), accumulates metabolic waste (e.g. lactate) and results in fatigue (Alexander, 1989; Goolish, 1991a; Sänger and Stoiber, 2001; Hedrick *et al.*, 2015). The fraction of the MS that is influenced by anaerobic metabolism is therefore not available for sustainable activity. Given that the OCLTT hypothesis concerns only aerobic performance (Pörtner, 2010), it is imperative that any anaerobic component of the MS is known and corrected for to ensure accurate predictions. Although previous studies have acknowledged an anaerobic component within MS (Goolish, 1991a; Reidy *et al.*, 2000; Svendsen *et al.*, 2012; Careau *et al.*, 2014; Norin *et al.*, 2014), the component has, to date, received little quantitative attention in relation to OCLTT.

Fish acquire energy to accomplish different types of physiological work, such as biosynthesis (e.g. somatic growth), maintenance (e.g. circulation, respiration and osmoregulation) and generation of external work to allow locomotion (Careau *et al.*, 2014). If all these functional traits were running at maximal rate, the oxygen requirements would exceed the available supply, forcing individual organisms to prioritize their oxygen budget within the finite size of MS (Killen *et al.*, 2007; Guderley and Pörtner, 2010; Holt and Jørgensen, 2015). For example, the metabolic costs of locomotion and digestion exhibit a trade-off in several fish species (Priede, 1985; Jordan and Steffensen, 2007; Altimiras *et al.*, 2008; Li *et al.*, 2010), which has ecological and evolutionary implications in relation to important performance traits (e.g. predator evasion, foraging, growth, migration and reproduction; Reidy *et al.*, 2000; Oufiero and Garland, 2009). In some fish species, however, metabolism associated with digestion may be additive to the metabolism associated with locomotion (Jourdan-Pineau *et al.*, 2010). Performance trade-offs often play important roles in relation to phenotypic variation found between individuals (Oufiero *et al.*, 2011; Seebacher and Walter, 2012; Svendsen *et al.*, 2015) and may take place when two antagonistic traits cannot be optimized simultaneously because of conflicting demands on the same capacity (Priede, 1985; Roff and Fairbairn, 2007; Svendsen *et al.*, 2015), such as the oxygen budget (Farrell, 2007; Altimiras *et al.*, 2008). This implies that excellence in one trait comes at the cost of performance in a different trait (Vanhooydonck *et al.*, 2014; Walker and Caddigan, 2015), which is classically exemplified by the conflicting relationship between sprinters and endurance athletes (Reidy *et al.*, 2000; Van Damme *et al.*, 2002; Marras *et al.*, 2013). To date, evidence of the corresponding locomotory trade-off in fishes remains inconclusive, with some studies finding support (Reidy *et al.*, 2000; Ojanguren and Braña, 2003; Langerhans, 2009; Oufiero *et al.*, 2011; Ellerby and Gerry, 2011; Yan *et al.,* 2012), whereas others have not (Claireaux *et al.*, 2007; Oufiero and Garland, 2009; Seebacher and Walter, 2012; Marras *et al.*, 2013).

In many fish species, locomotor performance is powered by the myotomal musculature, consisting of segmented red and white muscle fibres. Red oxidative muscles are slow contracting, fuelled by aerobic metabolism and power steady, sustainable swimming (Webb, 1993, 1998; Kieffer, 2000; Sänger and Stoiber, 2001). When approaching swimming speeds that exceed the power capacity and contraction speed of the red muscle fibres, a gait transition to unsteady, unsustainable, burst-assisted swimming occurs with the activation of fast white muscle fibres. White fibres are mainly fuelled by anaerobic metabolism, and their activation depletes substrates (e.g. glycogen), accumulates metabolic waste (e.g. lactate) and results in fatigue (Webb, 1993; Kieffer, 2000; Sänger and Stoiber, 2001).

Anaerobic metabolism may occur at submaximal exercise levels (Goolish, 1991a; Svendsen *et al.*, 2010) and before reaching the maximal aerobic metabolic rate (Burgetz *et al.*, 1998; Lee *et al.*, 2003; Hinch *et al.*, 2006; Teulier *et al.*, 2013). Hence, the gait transition to burst-assisted swimming can be used to partition swimming performance and the MS (Peake and Farrell, 2004; Peake, 2008; Marras *et al.*, 2013; Svendsen *et al.*, 2015) into a sustainable aerobic component and an unsustainable component strongly influenced by anaerobic metabolism. While trade-offs related to sustainable (aerobic) and unsustainable (anaerobic) swimming performances have been examined (Ellerby and Gerry, 2011; Yan *et al.*, 2012; Marras *et al.*, 2013), to date no study has tested for trade-offs related to sustainable and unsustainable components of the MS.

Based on existing data on fish swimming performance and metabolism (Svendsen *et al.*, 2013, 2015), the present study examined the OCLTT assumption that the fraction of the MS which is influenced by anaerobic metabolism, is insignificant. At increasing swimming speeds, the gait transition speed (*U*_GT_) to burst-assisted swimming was used to partition swimming performance into a sustainable and strictly aerobic component (*U*_sus_) and an unsustainable component influenced by anaerobic metabolism (*U*_unsus_). This partitioned MS into the MS associated with sustainable swimming speeds ≤ *U*_GT_ (sustainable metabolic scope; MS_sus_) and the MS associated with unsustainable swimming speeds > *U*_GT_ (unsustainable metabolic scope; MS_unsus_). Using these data, we tested for trade-offs between the two swimming performance measures (*U*_sus_ and *U*_unsus_) and between the two measures of the MS (MS_sus_ and MS_unsus_). We predicted negative correlations between both groups of measures, implying that individuals cannot optimize *U*_sus_ and *U*_unsus_ or MS_sus_ and MS_unsus_ simultaneously.

## Materials and methods

### Animals

A total of 13 gilthead sea bream (*Sparus aurata*; unknown sex; mean ± SEM body mass 79.8 ± 2.4 g and length 14.8 ± 0.2 cm) from a ﬁsh farm (Ferme Marine de Douhet) in France were maintained in a holding tank (0.7 m^3^) with seawater (salinity of 30‰) at 10°C. In addition, 18 guppies (*Poecilia reticulata*; female) (body mass 0.296 ± 0.009 g and length 3.0 ± 0.0 cm) were captured in Trinidad and maintained in freshwater holding tanks (30 l) at 26°C. Fish were acclimated to the laboratory for at least 2 weeks and fed daily on commercial fish food.

### Respirometry

Two swimming respirometers (8.4 and 0.17 l) were used to measure oxygen consumption rate ($\dot{M}$O_2_; in milligrams of oxygen per kilogram per hour) as a function of swimming speed (*U*; in centimetres per second) in *S. aurata* and *P. reticulata*. Temperature-controlling instruments (TMP-REG; Loligo Systems, Tjele, Denmark) were employed to maintain temperatures at 10 and 26°C (±0.1°C), respectively. Oxygen partial pressure (in kilopascals) inside the respirometers was measured using ﬁbre-optic sensor technology (PreSens, Regensburg, Germany). Intermittent ﬂow respirometry (Forstner, 1983) was applied in accordance with previous studies (Steffensen, 1989; Peixoto *et al.*, 2016). The software AutoResp (Loligo Systems Aps, Tjele, Denmark) was used to collect data and calculate $\dot{M}$O_2_ from measurements of oxygen content inside the respirometers (Peixoto *et al.*, 2016).

### Experimental protocol

*Poecilia reticulata* were fasted for 24 h, whereas *S. aurata* were fasted for 48 h to ensure post-absorptive states and then transferred to the respirometers. Fish were acclimated to the respirometers for 8–12 h (overnight) while swimming at a speed of 0.5 body lengths (BL) s^−1^ (*S. aurata*) and 2 BL s^−1^ (*P. reticulata*) prior to collection of data. These speeds were the minimal swimming speeds that ensured positive rheotaxis. Critical swimming speed protocols were then used to measure $\dot{M}$O_2_ at increasing swimming speeds until fatigue (Brett, 1964; Svendsen *et al.*, 2013, 2015). The time interval at each swimming speed was 30 min for *S. aurata* and 12 min for *P. reticulata*, both including 15 s of speed increment to reach each new test speed.

**Figure 1:**
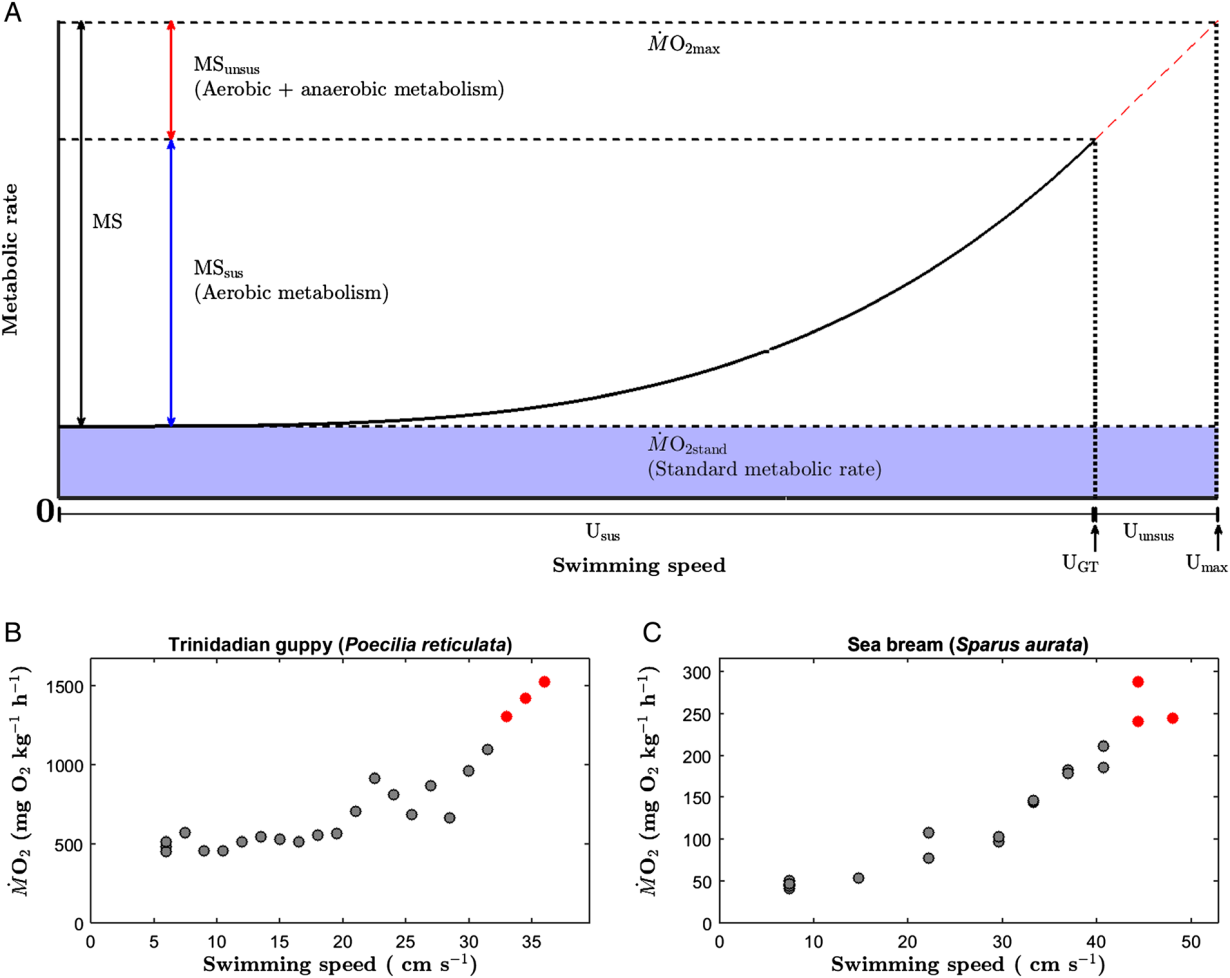
(**A**) Conceptual model and (**B** and **C**) raw data describing the metabolic rate as a function of swimming speed. (A) Schematic illustration showing the metabolic rate as function of swimming speed, including metabolic scope (MS; (black double-headed arrow) and gait transition speed (*U*_GT_) as the highest sustainable swimming speed, equivalent to *U*_STmax_ of Peake (2008). Using *U*_GT_, swimming performance is partitioned into sustainable (ranging from zero speed to *U*_GT_) and unsustainable (swimming speeds higher than *U*_GT_ until *U*_max_) components. The metabolic rate at *U*_GT_ is used to distinguish between sustainable metabolic scope (MS_sus_; blue double-headed arrow) and unsustainable metabolic scope (MS_unsus_; red double-headed arrow). (B and C) Raw data showing oxygen consumption rate ($\dot{M}$O_2_; in milligrams of oxygen per kilogram per hour) as a function of swimming speed (in centimetres per second) in an individual Trinidadian guppy (*Poecilia reticulata*; B) and gilthead sea bream (*Sparus aurata*; C) used in this study. Data are adapted from Svendsen *et al.* (2013, 2015). Grey symbols represent $\dot{M}$O_2_ when no burst-assisted swimming occurred, whereas red symbols represent $\dot{M}$O_2_ when burst-assisted swimming occurred.

Unlike steady swimming, burst-assisted swimming is partly fuelled by anaerobic metabolism (Peake and Farrell, 2004; Peake, 2008; Marras *et al.*, 2013). The number of bursts correlates positively with excess post-exercise oxygen consumption and therefore anaerobic metabolism (Svendsen *et al.*, 2010), suggesting that the onset of burst-assisted swimming can be used as a reliable indicator of anaerobic metabolism (Svendsen *et al.*, 2015). Hence, in parallel with the measurements of $\dot{M}$O_2_, the onset of burst-assisted swimming was recorded. The gait transition speed, *U*_GT_, was defined as the highest swimming speed that was supported using only a steady, undulatory locomotory gait (equivalent to *U*_STmax_ of Peake, 2008). The *U*_sus_ was defined as the range of sustainable swimming speeds between zero speed and maximal swimming speed maintained by steady swimming (*U*_GT_), whereas *U*_unsus_ was defined as the range of unsustainable swimming speeds higher than *U*_GT_ until the maximal swimming speed (*U*_max_; Fig. 1A).

### Data analyses

Applying data from individual fish, the relationships between *U* and $\dot{M}$O_2_ were described using an exponential equation (Brett, 1964; Fig. 1A). The analyses were limited to steady swimming speeds because the relationship between *U* and $\dot{M}$O_2_ tends to vary at speeds faster than *U*_GT_ (Schurmann and Steffensen, 1997; Svendsen *et al.*, 2013, 2015; Killen *et al.*, 2015). Extrapolating the exponential equation to zero speed provided an estimate of the standard metabolic rate ($\dot{M}$O_2stand_; Brett, 1964; McKenzie *et al*., 2003; Arnott *et al*., 2006; Fig. 1A). Maximal metabolic rate ($\dot{M}$O_2max_) was deﬁned as the highest $\dot{M}$O_2_ measured during the complete swimming protocol (i.e. until fatigue; Binning *et al.*, 2014). The MS was calculated as the difference between $\dot{M}$O_2max_ and $\dot{M}$O_2stand_ in individual fish (Fig. 1A).

The metabolic rate corresponding to maximal sustainable swimming speed was deﬁned as the maximal metabolic rate that was maintained aerobically at speeds up to, and including, *U*_GT_ without the accumulation of anaerobic metabolic products that contribute to performance (*U*) and negatively impact endurance (Hillman *et al.*, 2014). Hence, swimming performance and MS were partitioned into the following two different components: (i) sustainable components supported by aerobic metabolism alone (*U*_sus_ and MS_sus_); and (ii) unsustainable components strongly influenced by anaerobic metabolism (*U*_unsus_ and MS_unsus_; Fig. 1A).

To test for trade-offs in *S. aurata* and *P. reticulata* at the intraspecific level, components were correlated using least-squares linear regression (Zar, 2010). Regressions were multivariate, with the unsustainable components (i.e. *U*_unsus_ and MS_unsus_) as the dependent variables and the sustainable component (i.e. *U*_sus_ and MS_sus_) and body size (i.e. length and mass) as the independent variables. This was done to test whether predicted correlations were revealed independently of factors related to body size. Multivariate regressions were carried out using stepwise backward elimination. The trade-off related to swimming performance was tested by correlating *U*_sus_ and *U*_unsus_, whereas the trade-off related to the MS was tested by correlating MS_sus_ and MS_unsus_. In both cases, a trade-off would be revealed by a significant negative relationship.

In addition, we calculated the fractions (as percentages) of *U*_max_ and MS that were constituted by the unsustainable components (i.e. *U*_unsus_ and MS_unsus_, respectively). The unsustainable fractions (percentages) of *U*_max_ and MS were then correlated with the corresponding sustainable components (i.e. *U*_sus_ and MS_sus_). The correlations were tested using least-squares linear regressions.

Tests were carried out using the software MATLAB 8.5 (MathWorks, 2015), and results were considered significant at *P* < 0.05. All values are reported as means ± SEM unless otherwise stated.

## Results

### Burst-assisted swimming

**Figure 2:**
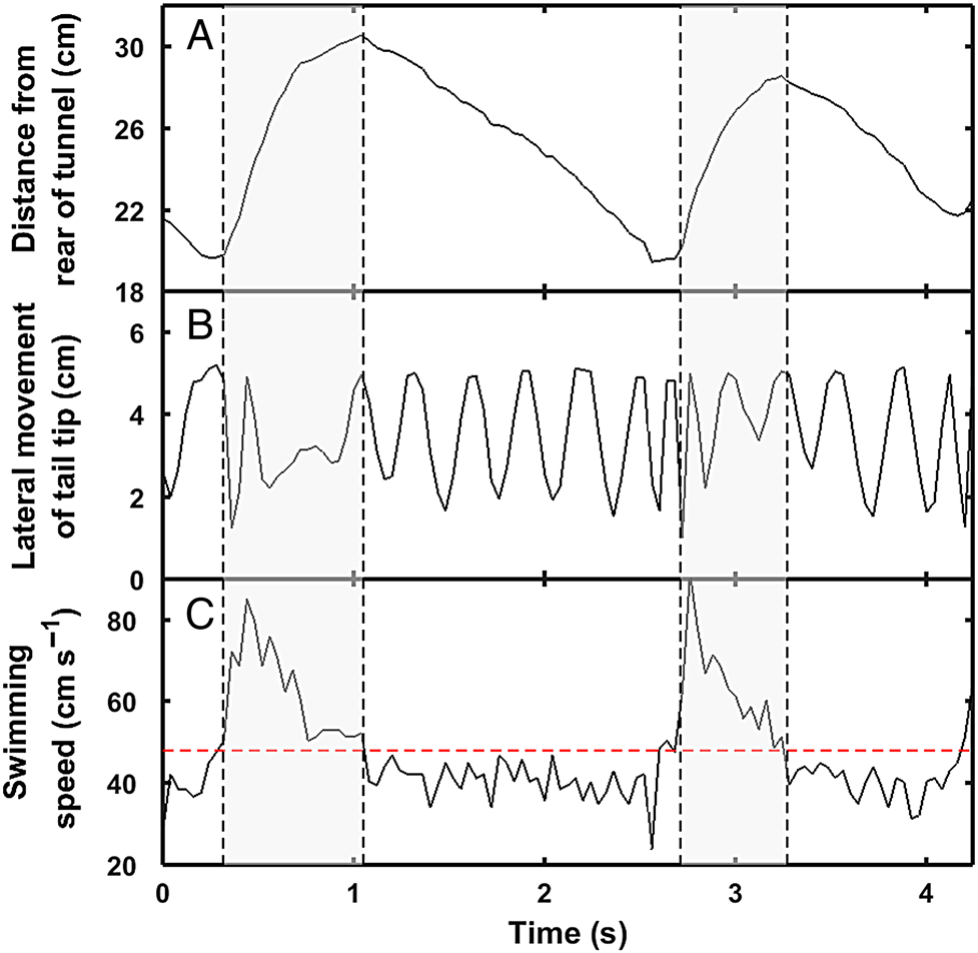
Burst swimming in gilthead sea bream (*S. aurata*; body length 14.5 cm), with bursts indicated using grey shading. Data were collected in a respirometry chamber [32 cm × 9 cm × 11 cm (length × width × height)], with the water speed adjusted to 48 cm s^−1^. (**A**) Longitudinal position of fish snout in the chamber. Bursts are associated with forward movement (grey shading) and followed by backwards movement in the chamber. The *y*-axis denotes the distance (in centimetres) from the most downstream end of the chamber. (**B**) Lateral movements of the tail tip as recorded dorsally, with increased tail beat frequency and amplitude providing thrust for each burst. (**C**) Fish swimming speed in the chamber, with peaks approaching 90 cm s^−1^ during bursts and speeds below the adjusted water speed (48 cm s^−1^; indicated by a dashed red line) after bursts. Note that the fish is moving backwards in the chamber (A) when the swimming speed is below 48 cm s^−1^ (C).

Burst-assisted swimming was evident as forward movement in the chamber (Fig. 2A), elevated tail beat frequency and amplitude (Fig. 2B) and increased swimming speed (Fig. 2C). Between bursts, fish swam slower than the test speeds (Fig. 2C), leading to backwards movement in the chamber (Fig. 2A) and subsequent bursts until fatigue. Burst-assisted swimming was observed in all fish, except three *P. reticulata*.

The value of *U*_max_ was 46.4 ± 1.5 cm s^−1^ (range, 37.1–57.0 cm s^−1^) and 44.0 ± 1.7 cm s^−1^ (range, 29.5–52.5 cm s^−1^) in *S. aurata* and *P. reticulata*, respectively. Relative to *U*_max_, *U*_unsus_ constituted 13.7 ± 1.9% (range, 6.7–27.3%) in *S. aurata* and 7.1 ± 1.2% (range, 0– 15.6%) in *P. reticulata*.

### Inconsistent correlations between sustainable and unsustainable swimming performances

**Figure 3:**
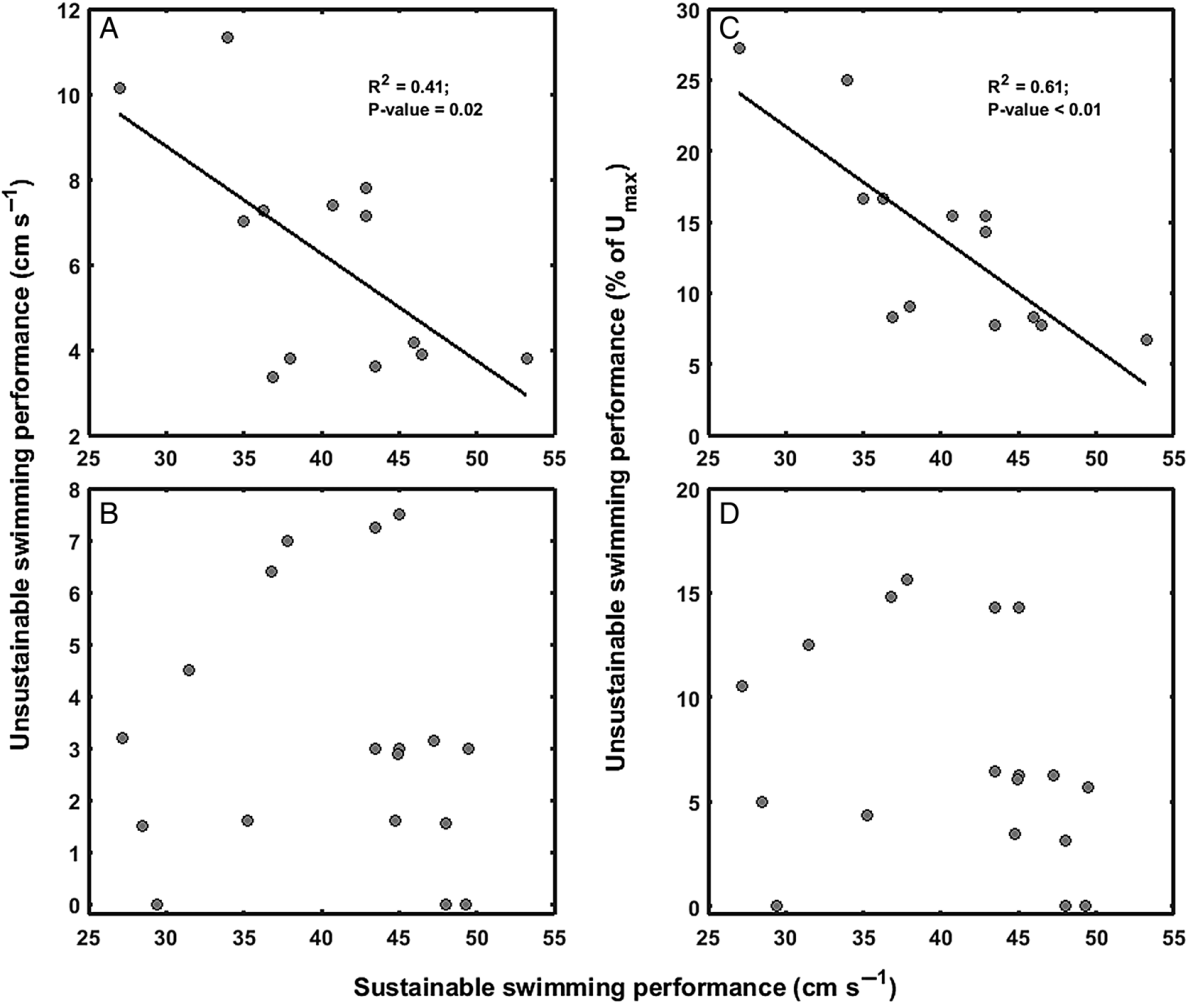
(**A** and **B**) Relationships between sustainable (*U*_sus_) and unsustainable (*U*_unsus_) swimming performances (see Fig. 1A for details). (**C** and **D**) Relationships between *U*_sus_ and the fraction (percentage) of *U*_max_ constituted by *U*_unsus_. Significant relationships for gilthead sea bream (*S. aurata*) were found between *U*_sus_ and *U*_unsus_ (A), and between *U*_sus_ and the fraction (percentage) of *U*_max_ constituted by *U*_unsus_ (C). (B and D) No significant relationships were found in Trinidadian guppy (*P. reticulata*).

Although a significant negative linear relationship between sustainable swimming performance (i.e. *U*_sus_) and unsustainable swimming performance (i.e. *U*_unsus_) was evident in *S. aurata* (*P* = 0.02, *R*^2^ = 0.41; Fig. 3A), no correlation was found for *P. reticulata* (*P* = 0.86, *R*^2^ = 0.002; Fig. 3B). For both species, the regression analyses indicated no effects of fish body length. The relationship between *U*_sus_ and the fraction (percentage) of *U*_max_ constituted by *U*_unsus_ was significant for *S. aurata* (*P* < 0.01, *R*^2^ = 0.61; Fig. 3C) but not for *P. reticulata* (*P* = 0.33, *R*^2^ = 0.06; Fig. 3D). Given that correlations were significant in only one species, these results suggested inconsistent relationships between sustainable and unsustainable swimming performances.

### Consistent negative correlation between sustainable and unsustainable metabolic scopes

**Figure 4:**
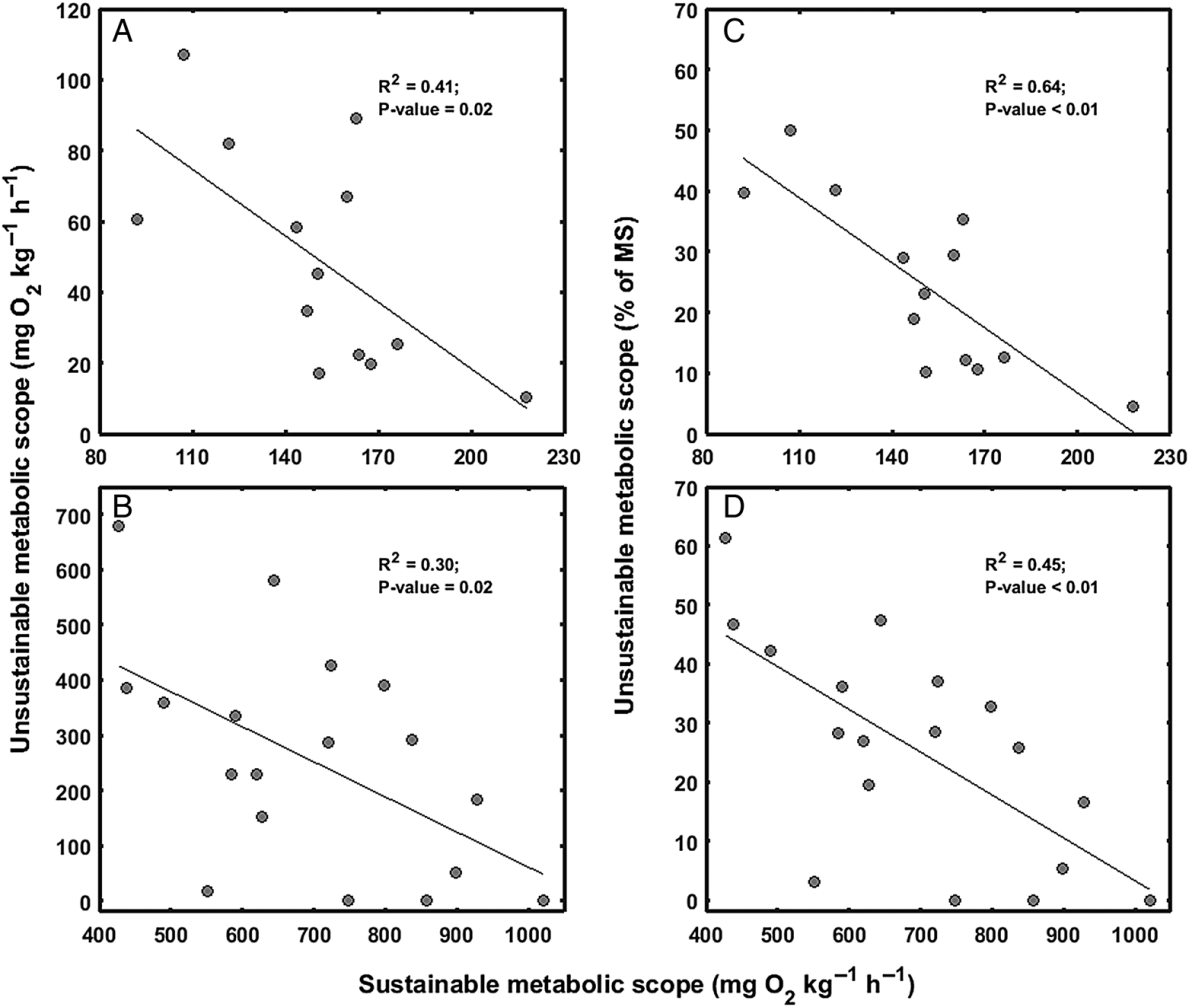
(**A** and **B**) Relationships between sustainable metabolic scope (MS_sus_) and unsustainable metabolic scope (MS_unsus_; see Fig. 1A for details). (**C** and **D**) Relationships between MS_sus_ and the fraction (%) of MS constituted by MS_unsus_, Significant negative relationships between MS_sus_ and MS_unsus_ or the fraction (percentage) of MS constituted by MS_unsus_ were found in both gilthead sea bream (*S. aurata*; A and C) and Trinidadian guppy (*P. reticulata*; B and D).

In addition to the trade-off in swimming performance, this study examined the trade-off between MS_sus_ and MS_unsus_ (Figs 1B and C and 4). The value of MS_unsus_ was 49.1 ± 8.6 mg O_2_ kg^−1^ h^−1^ (range, 10.3–107.2 mg O_2_ kg^−1^ h^−1^) and 255.1 ± 47.1 mg O_2_ kg^−1^ h^−1^ (range, 0–677.9 mg O_2_ kg^−1^ h^−1^) in *S. aurata* and *P. reticulata*, respectively*.* Relative to the MS, MS_unsus_ constituted 24.3 ± 3.9% (range, 4.5–50.0%) in *S. aurata* and 26.1 ± 4.2% (range, 0–61.3%) in *P. reticulata*. These findings indicated that significant fractions (means, 24.3 and 26.1%) of MS are influenced by anaerobic metabolism and not available for sustainable activities.

In contrast to the swimming performance data (Fig. 3), MS data revealed consistent significant negative correlations between MS_sus_ and MS_unsus_ in both *S. aurata* (*P* = 0.02, *R*^2^ = 0.41; Fig. 4A) and *P. reticulata* (*P* = 0.02, *R*^2^ = 0.30; Fig. 4B), indicating a trade-off where individuals exhibiting superior MS_sus_ exhibit inferior MS_unsus_. The regression analyses indicated no effects of body mass for either species. The relationships between MS_sus_ and the fraction (percentage) of MS constituted by MS_unsus_ were significant for both *S. aurata* (*P* < 0.01, *R*^2^ = 0.64; Fig. 4C) and *P. reticulata* (*P* < 0.01, *R*^2^ = 0.45; Fig. 4D).

## Discussion

Using two teleost species, this study tested for locomotory performance trade-offs between *U*_sus_ and *U*_unsus_; however, contrary to our prediction, we found no evidence of a consistent negative correlations. Nevertheless, data indicated significant trade-offs within the MS between MS_sus_ and MS_unsus_ in both species. Earlier studies have acknowledged that anaerobic metabolism may occur within the MS (Goolish, 1991b; Reidy *et al.*, 2000; Svendsen *et al.*, 2012; Norin *et al.*, 2014), but the fraction of the MS influenced by anaerobic metabolism remains largely unknown (Burgetz *et al.*, 1998; Farrell, 2007). The present study is the first to report intraspecific variation in MS_unsus_ in fish, and we found that MS_unsus_ may comprise up to 61% of MS in individual fish. This fraction of MS was associated with burst-assisted swimming and therefore partly fuelled by anaerobic metabolism. On average, MS_unsus_ constituted 24.3 and 26.1% of MS in *S. aurata* and *P. reticulata*, respectively, highlighting the importance of anaerobic metabolism within the MS. The fact that a significant fraction of the MS is influenced by anaerobic metabolism, and therefore not available for sustainable activity, could have important implications when MS is used to predict effects of environmental variation, particularly in relation to climate change and OCLTT.

### Inconsistent correlations between *U*_sus_ and *U*_unsus_

The negative correlation between *U*_sus_ and *U*_unsus_ observed in *S. aurata* indicates a locomotor performance trade-off. Conversely, we found no evidence for the same trade-off in *P. reticulata.* We recognize the fact that the significant correlation for *S. aurata* may be coincidental and may not hold true if a future study investigates the same relationship using a substantially increased sample size. Numerous studies have tested the conflicting nature of aerobic and anaerobic swimming performance, with equivocal results (Reidy *et al.*, 2000; Ojanguren and Braña, 2003; Claireaux and Lefrançois, 2007; Oufiero *et al.*, 2011; Seebacher and Walter, 2012; Yan *et al.*, 2012; Marras *et al.*, 2013). Reidy *et al.* (2000) found a negative relationship between aerobic and anaerobic swimming performance in Atlantic cod (*Gadus morhua*). This was supported by Oufiero *et al.* (2011), who presented evidence of a similar trade-off in Trinidadian killifish (*Rivulus hartii*). In contrast, Marras *et al.* (2013) did not detect a negative correlation between aerobic and anaerobic swimming performances in European sea bass (*Dicentrarchus labrax*). Comparing relationships between aerobic and anaerobic swimming performances across studies is difficult, because previous studies have employed a diversity of methods to determine swimming performance in many different species and in disparate environmental conditions (e.g. temperatures).

It has been argued that the spatial separation of red and white muscle fibres in most teleosts (Webb, 1993, 1998; Sänger and Stoiber, 2001; Johnston *et al.*, 2011) is consistent with the absence of a locomotor trade-off because the aerobic and anaerobic muscle structures are recruited separately (Claireaux *et al.*, 2007; Marras *et al.*, 2013). According to this conjecture, optimization of one type of muscle fibre does not come at cost to the other (Marras *et al.*, 2013). However, assuming morphological constraints, a greater proportion of red muscle fibres would presumably occupy the space available for white muscle fibres and thus, suppress anaerobic performance. Consequently, variation in the proportion of white muscle fibres could constitute the mechanistic basis of negative relationships between aerobic and anaerobic swimming performances in individual fish.

Although metabolism is important for swimming performance (Priede, 1985; Binning *et al.*, 2015), physiology is not the sole determinant of swimming performance. Several intraspecific studies have shown that swimming performance may be unaffected by variation in metabolism (Anttila *et al.*, 2014; Svendsen *et al.*, 2015) and strongly affected by morphology (Drucker and Lauder, 2000; Domenici *et al.*, 2008; Langerhans, 2009) and biomechanics (Langerhans, 2009; Shadwick and Goldbogen, 2012; Svendsen *et al.*, 2013). Consequently, intraspecific variation in morphology and biomechanics may mask physiological variation (and trade-offs) and could explain the inconsistent correlations between *U*_sus_ and *U*_unsus_ observed in the present study.

### Consistent negative correlations between MS_sus_ and MS_unsus_

Although this study provides inconsistent evidence for a trade-off related to swimming performance, our data show unambiguous support for a physiological trade-off between MS_sus_ and MS_unsus_ in two teleost species, suggesting that MS_sus_ and MS_unsus_ are two antagonistic traits that cannot be optimized simultaneously.

The mechanistic basis for the apparent trade-offs between MS_sus_ and MS_unsus_ or *U*_sus_ and *U*_unsus_ remain uncertain and poorly described. However, a general prediction is that a functional trade-off exists between sustainable and unsustainable locomotion for fishes using a coupled locomotor system, where the same body parts are used for propulsion in both sustainable and unsustainable locomotion (Yan *et al.*, 2012). The red muscles are oxidative tissues and cannot function without the supply of oxygen in order to yield ATP (Webb, 1998; Seebacher and Walter, 2012). The supply of oxygen to sustain aerobic activity may depend on the ability of the fish to extract oxygen from the water (Priede, 1985; Davison, 1997; Sänger and Stoiber, 2001) and cardiac performance (Farrell, 2007; Eliason *et al.*, 2011; Eliason and Farrell, 2015). In fact, it has been proposed that *U*_GT_ is limited by the oxygen supply to the heart rather than to the red muscles themselves, implying that fish with superior cardiac performance also exhibit higher *U*_GT_ (McKenzie and Claireaux, 2010). The present study used the recruitment of white muscles (i.e. gait transition) to partition MS into MS_sus_ and MS_unsus_, suggesting that MS_sus_ could also be limited by the capacity of the red muscles (e.g. contraction speed; Alexander, 1989; Johnston and Altringham, 1991; Drucker and Lauder, 2000) and not by the oxygen supply alone. The contractile properties of the red muscle fibres may determine a peak power production corresponding to the maximal swimming speed that can be achieved before the white muscles are recruited (i.e. *U*_GT_; Drucker and Lauder, 2000). For example, if a fish is constrained by the contraction speed of its red muscles, it may recruit the white muscles at a slow swimming speed, even if the oxygen supply is sufficient. Jayne and Lauder (1994) found that white muscles are recruited before the oxidative capacity of the red muscles is fully exploited, which potentially leaves an aerobic component within the red muscle to be used during unsustainable locomotion (i.e. burst-assisted swimming). Given that red muscles may remove catabolites produced in the white muscles (Wittenberger and Diaciuc, 1965; Johnston and Moon, 1980; Milligan and Girard, 1993; Richards *et al.*, 2002), unexploited aerobic capacity would be available to metabolize anaerobic waste products and elevate MS_unsus_. Hence, if a fish is using only 70% of the red muscle capacity when the white muscles are recruited, then it would have a substantial aerobic component (30%) within the red muscles to metabolize lactate from unsustainable swimming. Consequently, supporting our findings, anaerobically influenced performance (i.e. *U*_unsus_ and MS_unsus_) would be enhanced at the expense of aerobic performance (i.e. *U*_sus_ and MS_sus_) and *vice versa*.

The fate of lactate, however, is not determined by the aerobic capacity of the red muscles alone, and other oxidative tissues, such as the liver, gills and heart (Milligan and Farrell, 1991; Milligan and Girard, 1993; Milligan, 1996; Omlin and Weber, 2013), may also contribute to the metabolism of lactate. Yet, studies have indicated that white muscles have a limited ability to export lactate in rainbow trout (*Oncorhynchus mykiss*; Weber, 1991; Omlin and Weber, 2013) and suggest that the separation of red and white muscle structures precludes intramuscular lactate shuttles (Weber, 1991; Teulier *et al.*, 2013). If so, the suggestion that limiting contractile properties of the red muscles may leave an aerobic fraction for removal of anaerobic waste products would not underpin the metabolic trade-off observed in the present study.

Assuming that MS_sus_ most directly depends on red muscles, expansion of the aerobic capacity presumably involves increasing the proportion of red muscles and the mitochondrial content of individual fibres (Johnston and Altringham, 1991; Wieser, 1995). Red muscle fibres comprise 10–30 times higher volume densities of mitochondria compared with white muscle fibres (Johnston and Altringham, 1991; Sänger and Stoiber, 2001; Moyes and Genge, 2010), implying that the ratio between the two fibre types could influence the magnitude of MS_sus_ and MS_unsus_, because aerobic production of ATP takes place in the mitochondria (Johnston and Altringham, 1991; Sänger and Stoiber, 2001). This suggests that a fish with a bigger proportion of red muscle fibres could have more mitochondria per body volume, hence a greater MS_sus_ (Moyes and Genge, 2010). However, it has been argued that the volume density of mitochondria is not always a reliable descriptor of the aerobic potential, owing to differences in mitochondrial cristae density and aerobic enzyme activity (Johnston and Altringham, 1991). Instead, several studies have scaled the content of mitochondrial and glycolytic (i.e. anaerobic) enzymes in relation to body size, and although exceptions exist (Siebenaller *et al.*, 1982; Norton *et al.*, 2000), data have revealed negative scaling relationships between the two types of enzymes (Childress and Somero, 1990; Wieser, 1995; Moyes and Genge, 2010), supporting our findings of a metabolic trade-off between MS_sus_ and MS_unsus_. Nevertheless, the mechanistic basis of the apparent trade-off between MS_sus_ and MS_unsus_ within the MS needs further attention before firm conclusions can be drawn (Crans *et al.*, 2015).

### Fish behaviour in swim tunnels

Since the early work of Brett (1964), swim tunnels have become widely used tools to elucidate patterns of hydrodynamics (Liao, 2007), swimming performance (Schurmann and Steffensen, 1997; Tudorache *et al.*, 2007, 2010) and physiology (Peake and Farrell, 2004; Clark *et al.*, 2013; Svendsen *et al.*, 2013, 2015) of fishes. However, the ecological relevance of swimming performance measures obtained from forced swimming experiments has been questioned because the uniform hydraulic conditions in swim tunnels may be rare in nature (Kemp *et al.*, 2011; Maddock *et al.*, 2013). Instead, spontaneously moving fish perform frequent changes in speed and direction (Tudorache *et al.*, 2009; Steinhausen *et al.*, 2010). Importantly, fish swimming behaviour may be influenced by tunnel dimensions (Tudorache *et al.*, 2007), suggesting that fish swimming behaviour could have been influenced by the tunnel designs used in the present study. It is possible that the correlations between MS_sus_ and MS_unsus_ observed here are partly explained by behavioural responses to the swim tunnels and perhaps not relevant in natural settings. Likewise, it is not known whether intraspecific variation in MS_unsus_ is dependent on the protocol used for data collection. It is possible that the chase protocol to estimate MS (Reidy *et al.*, 2000; Clark *et al.*, 2013; Gräns *et al.*, 2014) would have resulted in less variation between individuals, because this methodology might be less affected by behavioural differences between individuals, including behavioural responses to a swim tunnel. The present study used swimming respirometry because this methodology typically provides the highest measures of $\dot{M}$O_2max_ (Roche *et al.*, 2013).

### Spontaneous use of metabolic scope

How frequently are fish spontaneously using 100% of their MS? Although the answer to this question is largely unknown, previous studies have indicated that this metabolic level may be engaged rarely (Priede, 1985; Lucas *et al.*, 1993; Murchie *et al.*, 2011; Genz *et al.*, 2013; Marras *et al.*, 2013). For example, Lucas *et al.* (1993) found that the northern pike (*Esox lucius*) rarely works at the upper limits of metabolism in the wild. Likewise, Murchie *et al.* (2011) documented that the Bahamas bonefish (*Albula vulpes*) typically operates at metabolic rates between 40 and 60% of the MS. It is unclear why fish rarely exploit the full extent of their metabolic capacity; however, it is possible that fish refrain from a level of aerobic metabolism that also incurs anaerobic metabolism. The present study found that on average, anaerobic metabolism was present in ∼25% of the MS. Given that the use of anaerobic metabolism is highly inefficient (Johnston and Moon, 1980; Goolish, 1991b), curtails prolonged locomotor performance (Reidy *et al.*, 2000) and is energetically expensive to recover from (Goolish, 1991b; Lucas *et al.*, 1993; Lee *et al.*, 2003; Svendsen *et al.*, 2010), it is likely that fish minimize the use of the MS that includes anaerobic metabolism (Goolish, 1991a; Lucas *et al.*, 1993). This highlights the importance of accounting for MS_unsus_ when the MS is used to estimate the effects of environmental stressors (e.g. temperature and hypoxia) on fish physiology and performance.

### Relevance for the oxygen- and capacity-limited thermal tolerance hypothesis and conservation physiology

In the present study, MS_unsus_ occupied between 0 and 61% of the MS in different individuals, suggesting that sustainable metabolic performance may differ substantially between two individuals, even if they exhibit similar MS. Likewise, Lee *et al.* (2003) indicated that the onset of burst swimming occurred at between 59 and 62% of the critical swimming speed in two salmon species (*Oncorhynchus nerka* and *Oncorhynchus kisutch*), leaving ∼40% of the swimming capacity influenced by anaerobic metabolism. Likewise, Burgetz *et al.* (1998) measured anaerobic metabolism at 70% of the critical swimming speed based on fuel store depletion and accumulation of anaerobic waste. Although the exact contribution of anaerobic metabolism to the total energy consumption (e.g. ATP synthesis rate) remains unknown, the mean percentage (25%) of the MS influenced by anaerobic metabolism observed in the present study still emphasizes the importance of MS_unsus_ when assessing the sustainable component of the MS.

The MS of individual fish is often translated into capacities for fitness-related performances (e.g. growth and locomotion; Guderley and Pörtner, 2010; Eliason *et al.*, 2011; Khan *et al.*, 2014) and related to habitat use (Claireaux and Lefrançois, 2007; del Raye and Weng, 2015) and migratory patterns (Cooke *et al.*, 2013; Eliason *et al.*, 2013) in a number of fish species. Therefore, MS provides an important tool within the field of conservation and climate change management because it acts as a filter between environmental conditions and impacts on population level (Farrell *et al.*, 2008; Jørgensen *et al.*, 2012; Seebacher and Franklin, 2012). For example, the concept of OCLTT has rapidly gained popularity within climate change research of ectotherms, and it hypothesizes that MS, which is considered an aerobic capacity, can be used to predict how local effects of environmental conditions will affect the physiology of fish (Pörtner and Farrell, 2008; Pörtner and Peck, 2010). However, while OCLTT is applied to estimate the physiological persistence of fish, no study measuring MS in relation to the OCLTT hypothesis has, to our knowledge, accounted for the impact of anaerobic metabolism (Cucco *et al.*, 2012; MacMillan *et al.*, 2012; Seth *et al.*, 2013; Norin *et al.*, 2014; Holt and Jørgensen, 2015), although it may be prevalent within the MS as demonstrated by the present study. Therefore, the assumption that the MS, assessed as the difference between $\dot{M}$O_2max_ and $\dot{M}$O_2stand_, is purely aerobic and available for sustainable activities (Reidy *et al.*, 2000; Clark *et al.*, 2013; Farrell, 2016) may be questioned. To improve conservation physiology of fishes, studies of MS in relation to environmental stressors and OCLTT may reveal better predictive value if they take into account that a significant part of MS is influenced by anaerobic metabolism and is unavailable for sustainable performances, as suggested by the present study. For instance, the relationship between temperature and MS_sus_ might differ from the relationship between temperature and MS, although this hypothesis is yet to be to be tested. Comparing normoxic and hypoxic treatments, Dutil *et al*. (2007) found that MS_unsus_ declines in hypoxia whereas MS_sus_ remains unchanged at least down to 50% air saturation, indicating that MS_unsus_ and MS_sus_ are not necessarily affected equally by environmental variation. The possibility remains that temperature variation affects MS_sus_ and MS_unsus_ differently, warranting further study of aerobic and anaerobic metabolism across environmental temperatures in relation to the OCLTT.

## Funding

This research was supported by a grant (SFRH/BPD/89473/2012) from the Foundation for Science and Technology (FCT) in Portugal to J.C.S., and by the Danish Council for Strategic Research project SUNFISH (sustainable fisheries, climate change and the North Sea ecosystem; grant no. 09-063096). Finally, this study was partly supported by Department of Biology at the University of Copenhagen (grant no. 102-0218/11-5550) and by the Aalborg Zoo Conservation Foundation.
